# Oxaliplatin combined with capecitabine therapy and comprehensive nursing in advanced colorectal cancer patients

**DOI:** 10.3389/fmed.2025.1582683

**Published:** 2025-07-02

**Authors:** Dan Kuang, Hong Xu, Xiaoming Shen

**Affiliations:** ^1^Department of Nursing, Affiliated Hospital of Jiangnan University, Wuxi, China; ^2^Department of Oncology, Affiliated Hospital of Jiangnan University, Wuxi, China; ^3^Department of Gastrointestinal Surgery, Affiliated Hospital of Jiangnan University, Wuxi, China

**Keywords:** colorectal cancer, oxaliplatin, capecitabine, chemotherapy, comprehensive nursing

## Abstract

**Objective:**

This study aimed to discuss the influence of oxaliplatin combined with capecitabine therapy and comprehensive nursing in advanced colorectal cancer (CRC) patients.

**Methods:**

A total of 100 advanced CRC patients, selected from January 2019 to January 2021, were divided into a control group (CG) and a research group (RG). Patients in the CG received capecitabine tablets and underwent routine nursing, while those in the RG received capecitabine tablets along with intravenous infusion of oxaliplatin and underwent comprehensive nursing. The short-term effects, levels of tumor markers, immune function, quality of life, occurrence of adverse reactions, negative emotions, and long-term survival rate in both groups were compared.

**Results:**

In comparison to the CG, the RG exhibited a higher total effective rate (*p* < 0.05), lower cancer antigen 125 (CA125) and carbohydrate-associated antigen 199 (CA199) levels (*p* < 0.05), higher CD4^+^ and CD4^+^/CD8^+^ levels (*p* < 0.05), a higher Karnofsky performance status (KPS) score, a lower occurrence of adverse reactions (*p* < 0.05), lower Self-Rating Anxiety Scale (SAS) and Self-Rating Depression Scale (SDS) scores (*p* < 0.05), and a better long-term survival rate of patients after 2 years of follow-up (*p* < 0.05).

**Conclusion:**

Oxaliplatin combined with capecitabine therapy, with comprehensive nursing, can effectively reduce clinical symptoms, enhance the immune function, and improve the quality of life of advanced CRC patients.

## 1 Introduction

Colorectal cancer (CRC) is a common type of malignant tumor in the digestive system, often occurring in the colon and rectum (1). The main cause of CRC is mostly related to environmental factors, dietary habits, and genetics (2). Patients with early-stage CRC usually do not have obvious symptoms. The occurrence and development process of CRC is very insidious, and it is not until the disease progresses to an advanced stage that patients gradually experience symptoms such as changes in bowel habits, bloody stools, abdominal pain, and abdominal masses. In severe cases of CRC, the cancer could also spread to the surrounding or distant tissues, thereby significantly shortening the survival period of the patients (3). At present, surgery is the main treatment for patients with early-stage CRC, while chemotherapy is advised for advanced CRC patients (4). The two chemotherapy regimens, FOLFOX (leucovorin + 5-fluorouracil + oxaliplatin) and FOLFIRI (leucovorin + 5-fluorouracil + irinotecan), are the first-line treatment options for advanced CRC patients (5, 6). However, the FOLFOX regimen requires continuous intravenous infusion of 5-fluorouracil through a central venous indwelling catheter, which could lead to phlebitis or thrombosis (7). In the FOLFIRI regimen, irinotecan exerts its anti-tumor effect by inhibiting topoisomerase I. However, it may cause severe diarrhea and myelosuppression, and its tolerance is lower in elderly patients and those with poor physical conditions (8).

Capecitabine is a chemotherapy drug that is widely used in the treatment of advanced CRC (9). It is an anti-metabolic fluoropyrimidine deoxyriboside carbamate that can be converted into 5-fluorouracil in the human body. Capecitabine has a targeted effect on cancer cells by effectively influencing the DNA synthesis of cancer cells and inhibiting their activity (10). However, the overall therapeutic effect of capecitabine when used alone is not satisfactory (11). Oxaliplatin belongs to the third generation of water-soluble platinum-based anticancer drugs. This drug interferes with the DNA synthesis process by producing hydrating derivatives, thus inhibiting tumor cell activity and affecting tumor cell division, with a significant anticancer effect and low cytotoxicity (12). It has been indicated that the combination of oxaliplatin and capecitabine can enhance the anti-tumor effect (13). Capecitabine has the potential to replace 5-fluorouracil/leucovorin as the optimal combination partner for oxaliplatin at a higher cost (14). Multiple clinical studies have confirmed that the combination of oxaliplatin and capecitabine regimen has comparable efficacy to the FOLFOX regimen in advanced CRC, but with lower toxicity (15). Compared with the FOLFIRI regimen, the combination of oxaliplatin and capecitabine regimen has a lower incidence of neurotoxicity, and the bone marrow suppression and gastrointestinal reactions are relatively manageable (16).

Furthermore, patients undergoing chemotherapy are prone to adverse reactions, including bone marrow suppression, neurotoxicity, and gastrointestinal symptoms (17). Therefore, effective nursing measures should be adopted to relieve and reduce the symptoms of discomfort and promote more significant treatment outcomes (18).

This study was designed to investigate the influence of oxaliplatin combined with capecitabine therapy alongside comprehensive nursing care on advanced CRC patients.

## 2 Data and methods

### 2.1 General data

A total of 100 advanced CRC patients were selected from our hospital from January 2019 to January 2021. These patients were divided into a control group (CG) and a research group (RG) via a random number table method, with 50 patients in each group. The CG included 28 men and 22 women, aged between 40 and 75 years, with an average age of 55.35 ± 6.23 years. Among them, 25 patients were at stage III and 25 patients at stage IV. The RG consisted of 27 men and 23 women, aged between 39 and 74 years, with an average age of 55.42 ± 6.35 years. The group included 24 patients at stage III and 26 patients at stage IV. The general basic data of both groups presented no difference (*p* > 0.05). Our study was approved by the Ethics Committee of the Affiliated Hospital of Jiangnan University. All participants provided written informed consent before enrollment, and the study was conducted in accordance with the Declaration of Helsinki.

Inclusion criteria: (1) CRC was confirmed by pathological examination; (2) expected survival ≥3 months. Exclusion criteria: (1) Patients had received chemoradiotherapy in the last 30 days, (2) had other malignant tumors, (3) liver and kidney insufficiency, (4) diseases of the immune system and blood system with rheumatism, (5) gastrointestinal bleeding, and (6) patients allergic to capecitabine and oxaliplatin.

### 2.2 Sample size

The sample size was calculated using G*Power software (19). Assuming a *p*-value of less than 0.05 (two-tailed) as significant, and a power of 0.80, the calculated sample size was 100.

### 2.3 Randomization and blinding

A group randomization design was adopted for random grouping. The random allocation sequence was generated by a computer. The allocation confidentiality measures were achieved through sequential numbering, sealing, and opaque envelopes. Patients who met the inclusion criteria were randomly assigned to the CG and the RG in a 1:1 ratio. This was a single-blind study, with the participants being unaware of the allocation.

### 2.4 Therapeutic methods

After admission, both groups underwent routine tests, such as electrocardiogram (ECG), routine hematuria, and liver and kidney function tests.

Patients in the CG were treated with capecitabine tablets (Qilu Pharmaceutical Co., Ltd.) at a dose of 1,000 mg/m^2^ two times/d after 14 days of continuous use, which was then discontinued for 7 days and a 21-day course of treatment.

In addition to the capecitabine tablets, patients in the RG received intravenous infusion of oxaliplatin (Grand Pharmaceutical Huangshi Feiyun Pharmaceutical Co., Ltd). The process was as follows: Oxaliplatin at 130 mg/m^2^ was injected into 500 mL of 5% glucose solution for intravenous infusion for 3 ~ 4 h. The infusion began on the first day of capecitabine and was discontinued after 20–21 days for 1 course of treatment.

Both groups were treated for three courses.

### 2.5 Dosage adjustment principle

Doctors appropriately adjusted the dosage for the patients based on the severity of side effects. Generally, when the toxicity was at level 2 (such as moderate diarrhea), the dosage was reduced by 25%. When the toxicity was at a level of 3 to 4 (such as severe bone marrow suppression), the treatment was suspended until it returned to ≤ level 1, and then the dosage was reduced by 50%. The initial dosage reduction was typically limited to no more than 20% in order to avoid compromising the treatment efficacy.

### 2.6 Adherence with the prescribed plan

Capecitabine was prescribed to be taken for 2 weeks and then stopped for 1 week. Oxaliplatin was used on the first day of each 3-week treatment cycle. Patients strictly followed the medication schedule set by the doctor and were advised not to adjust the dosage or stop taking the medication on their own. Every day, the patients recorded the number of bowel movements, changes in the skin of their hands and feet, and body temperature. If they noticed fever (>38°C) or abnormal bleeding, they sought immediate medical attention. The patients recorded the severity of side effects in a personal diary and provided the information during follow-up visits to help the doctor assess the treatment effect and adjust the treatment plan. During chemotherapy, the patients regularly had routine blood and liver and kidney function tests. All side effects were closely monitored after chemotherapy, and the patients were provided with timely treatment. Doctors decided whether to adjust the chemotherapy plan or dosage based on the re-examination results and the patients’ physical condition.

### 2.7 Nursing methods

After admission, the patients in the CG underwent routine nursing. The nurse explained about tumors and chemotherapy to the patients. The nurse emphasized the common adverse reactions of chemotherapy and informed the patients of the specific measures to take when experiencing adverse reactions to the digestive system, such as nausea and vomiting, to help the patients remain mentally strong. At the same time, the nurse instructed the patients to maintain a light, high-nutrition, and high-calorie diet during chemotherapy. Emphasis was laid on the need to drink plenty of water and engage in moderate exercise to replenish the body’s energy needs and enhance the patient’s tolerance to chemotherapy. Regular disinfection and cleaning of the patients’ ward were supervised by the nurse.

The patients in the RG, after admission, additionally received comprehensive nursing, which included the following:

(1) Establishment of patient files: once the patient was admitted to the hospital, the nurse introduced the hospital environment and the attending doctor to the patient, and inquired in detail about the patient’s daily work, life, family situation, dietary habits, as well as the patient’s own and his/her family’s understanding of CRC and chemotherapy treatment methods. A comprehensive assessment was then conducted, and the patient’s file was established.

(2) Health education: the nurse distributed educational brochures to the patients and explained tumor pathogenesis, causes, treatment plans, chemotherapy methods, and common adverse reactions through a combination of oral explanations and visual materials.

(3) Psychological nursing: the nurse actively listened and communicated with the patient, understood the patient’s inner thoughts and feelings with respect and encouragement, and built a good nurse–patient relationship based on trust. The nurse also actively communicated with the patient’s family, guiding them to encourage and support the patient to reduce psychological pressure. During the chemotherapy break period, the nurse encouraged the patient to engage in recreational activities, such as writing, drawing, and listening to music, to divert attention and alleviate psychological stress. Each activity lasted 30 min.

(4) Pain nursing: during chemotherapy, patients can experience symptoms of pain at the site of the tumor, which can have an impact on the patients’ daily lives. For patients experiencing pain, the nurses not only administered painkillers as per the doctor’s instructions but also provided psychological interventions, such as playing soothing music, performing massages, applying cold or hot compresses, and conducting relaxation training to alleviate their pain. Each activity lasted 30 min.

(5) Nursing for adverse reactions caused by chemotherapy: during chemotherapy, patients are prone to many adverse reactions. The nurse provided targeted nursing based on the patients’ symptoms. The nurse advised patients experiencing nausea, vomiting, or diarrhea to appropriately increase their water intake while maintaining their daily intake between 2000 and 3,000 mL. Regular water intake helped minimize the irritation caused by chemotherapy drugs to the gastrointestinal mucosa, alleviate gastrointestinal symptoms, and prevent dehydration. For patients with bone marrow suppression, the nurse closely observed the patients’ physical condition and noted if there was a decrease in the red blood cell count. Patients with a significant decrease in red blood cells were instructed to avoid forceful coughing and defecation to reduce abdominal and thoracic pressure. Additionally, the nurses instructed the patients to perform actions such as getting up or squatting down slowly and gently. For patients with a decrease in white blood cells, the nurses regularly observed the areas on their bodies prone to infection, including the mouth, perineum, and perianal area, and instructed them to take a bath daily to maintain hygiene. The nurses carefully monitored the activities of patients with thrombocytopenia to lower their probability of falls and injuries. If the platelet count was lower than 20 × 10^9^/L, the patients were advised to have strict bed rest. Patients with hand-foot syndrome were instructed to take 100 mg of vitamin B6 tablets and 200 mg of vitamin C tablets orally, three times a day. At the same time, the nurse provided the patients with daily care, instructing them to keep their upper and lower limbs elevated with pillows when sleeping to facilitate the flow of venous blood. The nurse advised the patients to wash their hands and feet with warm water twice a day and apply non-irritating moisturizers, such as Vaseline and urea cream, to keep the skin moist and reduce peeling and cracking. Patients were advised to wear loose-fitting cotton socks and cotton clothes to avoid friction injuries. Patients with abnormal liver function were regularly monitored by the nurses. If their alanine transaminase (ALT)/aspartate transaminase (AST) levels increased by more than three times, chemotherapy was suspended, and liver-protective drugs were prescribed. For patients with cardiac toxicity, the nurses monitored their electrocardiogram, and when chest pain or breathing difficulties occurred, the risk of myocardial ischemia was evaluated.

(6) Nursing guidance after discharge: Nurses distributed chemotherapy instruction manuals to the patients and their family members. The manual included details about the chemotherapy cycle, the possible adverse reactions that may occur during chemotherapy, the corresponding measures to be taken, and contact information for the concerned doctors and nurses. On the other hand, based on the actual situation of the patients, the nurses formulated targeted and personalized care plans, which included psychological care intervention measures for the patients, nursing measures for those with adverse reactions during chemotherapy, and nursing measures for patients experiencing discomfort from the disease. To achieve follow-up management after the patients’ discharge, the nurses established a personal file for each patient and conducted regular follow-up visits to address the problems of the patients.

### 2.8 Observation indicators

(1) Therapeutic evaluation: based on the evaluation criteria for the efficacy of solid tumors formulated by the World Health Organization (WHO) (20), complete remission (CR) refers to the disappearance of lesions for over 1 month. Partial remission (PR) indicates that the lesion area has reduced by over 30% in 1 month. Stable disease (SD) indicates that the lesion area has decreased by no more than 30%. Progressive disease (PD) means the lesion area has increased by over 20% or new lesions have occurred. Clinical response rate = CR rate + PR rate.

(2) Cancer antigen 125 (CA125) and carbohydrate-associated antigen 199 (CA199) levels were determined by double-antibody sandwich enzyme-linked immunoassay (ELISA, Shenzhen Jingmei Biotechnology Co., Ltd.).

(3) The percentage of CD4^+^ and CD8^+^ levels was examined by flow cytometry, and the ratio of CD4^+^/CD8^+^ was calculated.

(4) The quality of life of patients was evaluated using the Karnofsky performance status (KPS) score (21). The KPS score ranged from 0 to 100, and a higher score reflected a better quality of life for patients.

(5) The occurrence of adverse reactions after treatment was analyzed, such as nausea, vomiting, myelosuppression, diarrhea, and hand-foot syndrome.

(6) The Self-Rating Anxiety Scale (SAS) and the Self-Rating Depression Scale (SDS) were implemented to measure the degree of negative emotions (22). A high score indicated severe negative emotions.

(7) The patients were followed up for 2 years, by home visit or telephone visit, and the survival of all patients was tracked.

### 2.9 Statistical analysis

The data were analyzed using SPSS 24.0 software. Measurement data were represented as (x ± s), and Student’s *t*-test was implemented for comparison. The statistical data were expressed in percent (%) and compared using the chi-squared (*χ*^2^) test. The *p-*value of <0.05 indicated that the difference was significant.

## 3 Results

### 3.1 Short-term effects in both groups

In comparison to the CG, the total effective rate of the RG was higher (*p* < 0.05, Table 1), suggesting that oxaliplatin combined with capecitabine therapy and comprehensive nursing could promote the short-term clinical effect in CRC patients.

**Table 1:** Short-term effects in both the control and research groups.

Groups	CR	PR	SD	PD	Total effective rate
Control group (*n* = 50)	15	10	16	9	25 (50.00%)
Research group (*n* = 50)	20	18	10	2	38 (76.00%)
*χ* ^2^					7.25
*p*					<0.05

### 3.2 Serum CA125 and CA199 levels in both groups

**Figure 1:**
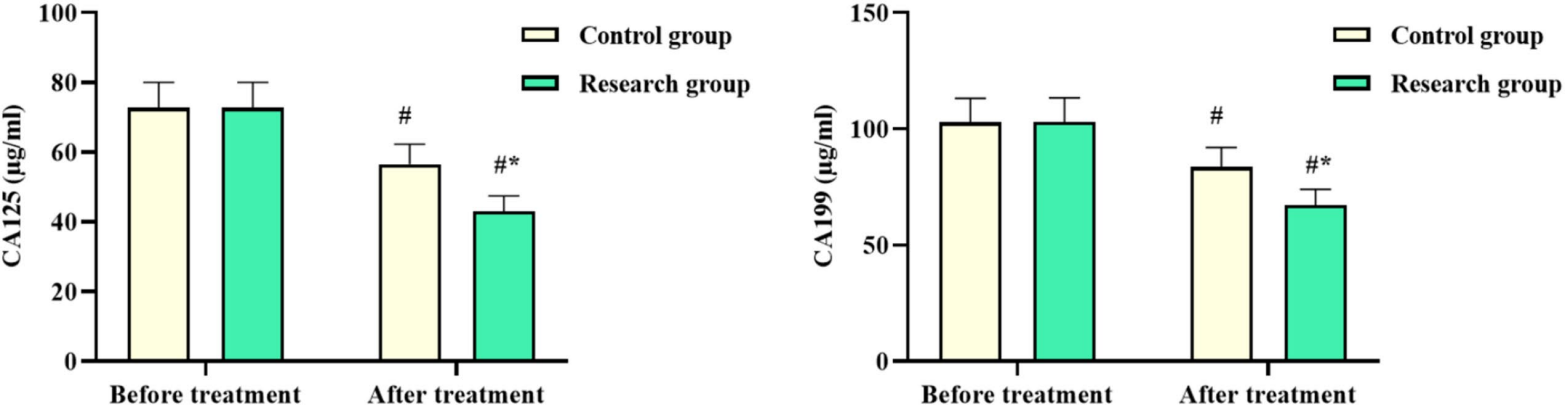
Serum CA125 and CA199 levels in the control and research groups. ^#^*p* < 0.05, compared with before chemotherapy. ^*^p < 0.05, compared to the control group.

No difference was observed in the CA125 and CA199 levels of both groups before chemotherapy (*p* > 0.05). The levels of CA125 and CA199 in both groups after chemotherapy declined, with the levels in the RG being lower than those in the CG (*p* < 0.05, Figure 1). These results suggested that oxaliplatin combined with capecitabine therapy and comprehensive nursing could inhibit tumor cell proliferation in CRC patients.

### 3.3 CD4^+^, CD8^+^, and CD4^+^/CD8^+^ levels in both groups

**Figure 2:**
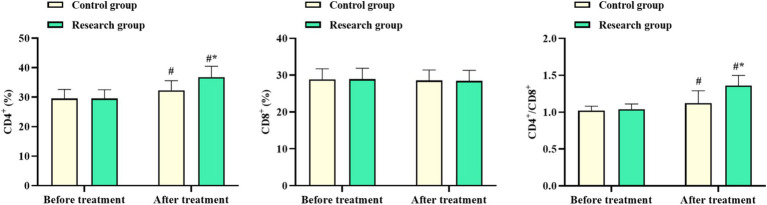
CD4^+^, CD8^+^, and CD4^+^/CD8^+^ levels in the control and research groups. ^#^*p* < 0.05, compared with before chemotherapy. ^*^p < 0.05, compared to the control group.

No difference was observed in CD4^+^ and CD4^+^/CD8^+^ levels of both groups before chemotherapy (*p* > 0.05). After chemotherapy, CD4^+^ and CD4^+^/CD8^+^ levels in the RG were elevated, with the levels in the RG being higher than in the CG. No differences were discovered in the CD8^+^ level between both groups before and after chemotherapy (*p* > 0.05, Figure 2). These results suggested that oxaliplatin combined with capecitabine therapy and comprehensive nursing could enhance the immune function of CRC patients.

### 3.4 KPS score in both groups

**Figure 3:**
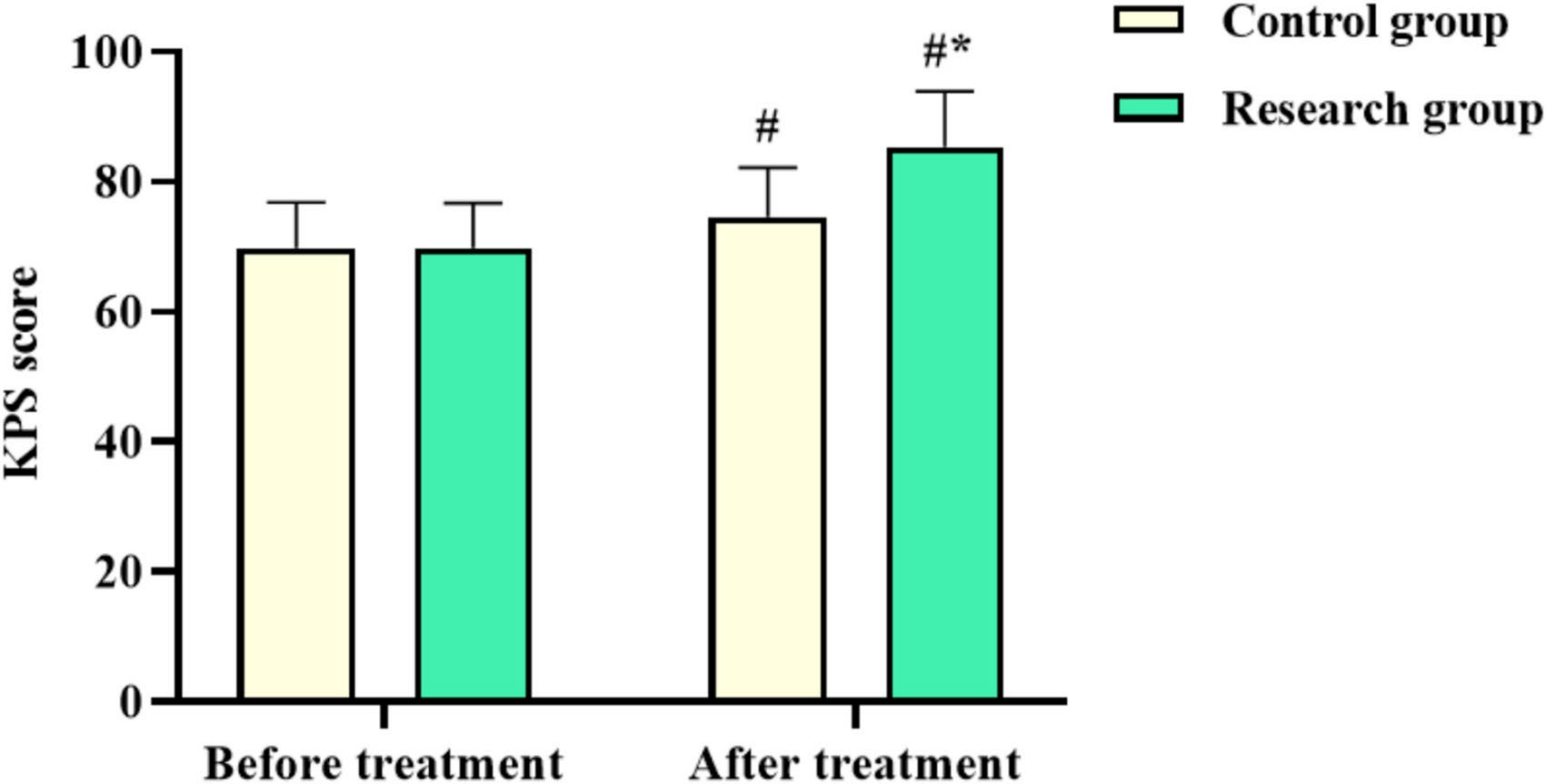
Karnofsky performance status (KPS) score in both groups. ^#^*p* < 0.05, compared with before chemotherapy. ^*^p < 0.05, compared to the control group.

No difference was observed in the KPS score of both groups before chemotherapy (*p* > 0.05). The KPS score in both groups after chemotherapy was elevated, with that in the RG being higher compared to the CG (*p* < 0.05, Figure 3). These results suggested that oxaliplatin combined with capecitabine therapy and comprehensive nursing could improve the quality of life of CRC patients.

### 3.5 Occurrence of adverse reactions in both groups

After chemotherapy, the occurrence of adverse reactions in the RG was lower compared to the CG (*p* < 0.05, Table 2).

**Table 2:** Occurrence of adverse reactions in both the control and research groups.

Groups	Nausea and vomiting	Myelosuppression		Diarrhea	Hand-foot syndrome	Total incidence rate
Control group (*n* = 50)	5	4		3	5	17 (34.00%)
Research group (*n* = 50)	2	2		1	2	7 (14.00%)
*χ* ^2^						5.48
*p*						<0.05

### 3.6 SAS and SDS scores in both groups

**Figure 4:**
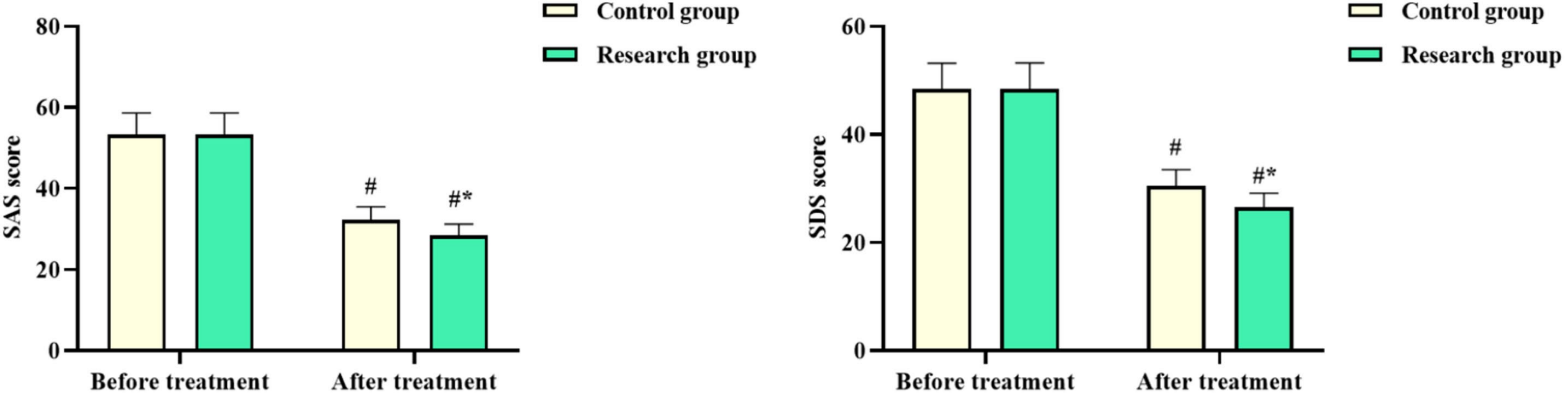
Self-Rating Anxiety Scale (SAS) and the Self-Rating Depression Scale (SDS) scores in both groups. ^#^*p* < 0.05, compared with before chemotherapy. ^*^p < 0.05, compared to the control group.

No difference was seen in the SAS and SDS scores of both groups before chemotherapy (*p* > 0.05). The SAS and SDS scores in both groups after chemotherapy declined, with those in the RG being lower compared to the CG (*p* < 0.05, Figure 4). These results suggested that oxaliplatin combined with capecitabine therapy and comprehensive nursing could relieve the degree of anxiety and depression in CRC patients.

### 3.7 Long-term survival rate in both groups

After 1 year of follow-up, no difference was observed in the long-term survival of both groups (*p* > 0.05). The long-term survival rate of patients in the RG after 2 years of follow-up was higher when compared to the CG (*p* < 0.05, Table 3).

**Table 3:** Long-term survival rate in both the control and research groups.

Groups	1 year of follow-up	2 years of follow-up
Control group (*n* = 50)	45 (90.00%)	28 (56.00%)
Research group (*n* = 50)	49 (98.00%)	44 (88.00%)
*χ* ^2^	2.84	12.70
*p*	>0.05	<0.05

## 4 Discussion

At present, surgery and multi-drug chemotherapy are the main treatment options for CRC (23). At the early stage of CRC, surgical treatment can provide satisfactory results. However, the early symptoms of CRC patients are not typical, and some even have no obvious symptoms. Therefore, most patients with CRC are in the advanced stage when seeking medical treatment, losing the best opportunity for treatment. Additionally, the effect of surgical treatment is poor. In this scenario, chemotherapy becomes important (24).

Capecitabine is an anti-metabolic fluoropyrimidine deoxyriboside carbamate that can be transformed into cytotoxic 5-fluorouracil *in vivo* (25). It can effectively inhibit the division of tumor cells, interfere with RNA and protein synthesis, and thus repress tumor cell proliferation. Additionally, capecitabine can reduce the damage caused by 5-fluorouracil to normal human cells (26). Oxaliplatin is a third-generation platinum-based anti-tumor drug, which mainly inhibits DNA synthesis and replication, inhibits tumor cell activity, affects cell division, and effectively kills tumor cells by binding to guanine (G) bases in DNA chains (27).

Furthermore, in the nursing of patients undergoing chemotherapy, the focus lies on the adverse reactions related to chemotherapy as well as mental health issues. Due to the patients’ lack of sufficient understanding of CRC and the chemotherapy process, they are prone to develop negative psychological emotions, such as anxiety, depression, and fear, which will further affect the effectiveness of the chemotherapy treatment (28). Comprehensive nursing is a comprehensive intervention model that focuses on the patient, meets the needs of patients during chemotherapy, and alleviates the pain caused by adverse reactions to chemotherapy (29).

The outcomes of this study showed that the remission rate of patients in the RG was higher than that in the CG, which reflects that the combination of capecitabine and oxaliplatin can enhance the therapeutic effect in CRC patients. The combination of the two drugs increased the content of 5-fluorouracil in tumor cells and targeted the inhibition of DNA replication and protein synthesis in tumor cells (30). In addition, oxaliplatin interferes with the replication and transcription of DNA, and along with capecitabine, synergistically inhibits the proliferation of tumor cells, which is significantly better than capecitabine alone (31). Consistently, Kibudde and Begg proposed that a drug combination of capecitabine and oxaliplatin showed a good overall response rate and survival in patients with metastatic CRC (32).

The ideal chemotherapy regimen can lessen the damage to the body’s immune function. CD4^+^/CD8^+^ is a biological index to determine whether the immune function is normal (33). The decrease of CD4^+^/CD8^+^ indicates that the immune function is low and is accompanied by immune function suppression (34). Besides, serum tumor markers have a crucial role in the diagnosis and prognosis assessment of CRC as non-invasive and effective screening methods (35). Among them, CA125 is expressed in various malignant tumors, and CA199, as a tumor-related antigen of the digestive tract, is highly expressed with the proliferation and differentiation of tumor cells (36). The outcomes of this study demonstrated that compared to the CG, the RG showed higher CD4^+^ and CD4^+^/CD8^+^ levels and lower CA125 and CA199 levels after chemotherapy. All these outcomes implied that capecitabine and oxaliplatin play a synergistic role in inhibiting tumor cell proliferation and improving immune function. The reason could be that capecitabine can enhance the DNA binding speed of oxaliplatin, while oxaliplatin can increase the activity of enzymes related to capecitabine action in tumor cells, and the combination of oxaliplatin and capecitabine can enhance the inhibitory effect on tumor cells (37). As reported previously, Park et al. (38) suggested that the combination of oxaliplatin and capecitabine was involved in the immune regulation of the liver metastatic CRC microenvironment via the cGAS-STING pathway. Lesterhuis et al. (39) discovered an enhanced non-specific T-cell reactivity upon oxaliplatin/capecitabine chemotherapy in colon cancer patients.

In addition, our study found that compared to the CG, the RG had a lower occurrence of adverse reactions, higher KPS score, and lower SAS and SDS scores, suggesting that comprehensive nursing could lessen the occurrence of adverse reactions, relieve the negative emotions, and also promote the quality of life of CRC patients undergoing oxaliplatin and capecitabine chemotherapy. In line with our findings, Li et al. (40) indicated that comprehensive nursing intervention could improve the quality of life of advanced prostate cancer patients undergoing chemoradiotherapy, increase their compliance with treatment, and reduce their adverse reactions, and therefore deserves clinical promotion. Schmoll et al. (41) suggested that capecitabine combined with oxaliplatin had a manageable tolerability profile in adjuvant therapy for stage III colon cancer. In addition, our study indicated that after 2 years of follow-up, the long-term survival rate of patients in the RG was better compared to that in the CG, reflecting that oxaliplatin combined with capecitabine can promote the long-term clinical efficacy of patients. Consistently, Bang et al. (42) suggested that 3-year disease-free survival was 74% in the oxaliplatin and capecitabine chemotherapy after surgery, and was considered a treatment option for patients with operable gastric cancer. Haller et al. (43) suggested that the addition of oxaliplatin to capecitabine improves disease-free survival in patients with stage III colon cancer.

Our research has some limitations. First, our sample size is relatively small, which could lead to deviations between the data results and the actual values. Second, our research is a single-center study, which could affect the general applicability of the research results to a broader population of CRC patients. Third, our research adopted a single-blind design, which inevitably led to subjective biases from the researchers, resulting in an imbalance in the treatment between the two groups. Fourth, although the report of the study showed a statistically significant improvement in the 2-year survival rate of the study group, the 2-year follow-up period may not be sufficient to evaluate the long-term survival benefits for patients with advanced CRC. Additionally, the tumor marker analysis shows significant reductions in CA125 and CA199 levels, but the study does not discuss how these biomarkers correlate with treatment response or long-term prognosis. Finally, our research did not analyze whether possible confounding factors, such as the distribution of patient tumor stages, previous treatments, comorbidities, or genetic factors, would affect the research results. Therefore, we will conduct a multivariate analysis of these variables, which will help determine whether the survival benefits are due to the treatment plan or other potential factors. At the same time, we will include a subgroup analysis that examines whether patients with greater reductions in CA125/CA199 have better survival outcomes. Besides, more multi-center, large-scale, and long-term clinical studies should be performed in the future to further validate our results.

## 5 Conclusion

Oxaliplatin and capecitabine chemotherapy combined with comprehensive nursing can effectively improve clinical symptoms, inhibit tumor cell proliferation, enhance immune function, reduce the occurrence of adverse reactions, enhance quality of life, and promote long-term clinical efficacy in advanced CRC patients. Our findings may provide additional adjuvant treatment and nursing options for advanced CRC patients.

## Data Availability

The raw data supporting the conclusions of this article will be made available by the authors, without undue reservation.
